# Regulation of HTLV-1 transformation

**DOI:** 10.1042/BSR20211921

**Published:** 2022-03-11

**Authors:** Kyle J. Ernzen, Amanda R. Panfil

**Affiliations:** 1 Department of Veterinary Biosciences, College of Veterinary Medicine, The Ohio State University, Columbus, OH, U.S.A.; 2 Center for Retrovirus Research, The Ohio State University, Columbus, OH, U.S.A.

**Keywords:** Hbz, HTLV-1, oncogenesis, Tax, transformation

## Abstract

Human T-cell leukemia virus type 1 (HTLV-1) is the only identified oncogenic human retrovirus. HTLV-1 infects approximately 5–10 million people worldwide and is the infectious cause of adult T-cell leukemia/lymphoma (ATL) and several chronic inflammatory diseases, including HTLV-1-associated myelopathy/tropical spastic paraparesis (HAM/TSP), dermatitis, and uveitis. Unlike other oncogenic retroviruses, HTLV-1 does not capture a cellular proto-oncogene or induce proviral insertional mutagenesis. HTLV-1 is a trans-activating retrovirus and encodes accessory proteins that induce cellular transformation over an extended period of time, upwards of several years to decades. Inarguably the most important viral accessory protein involved in transformation is Tax. Tax is a multifunctional protein that regulates several different pathways and cellular processes. This single viral protein is able to modulate viral gene expression, activate NF-κB signaling pathways, deregulate the cell cycle, disrupt apoptosis, and induce genomic instability. The summation of these processes results in cellular transformation and virus-mediated oncogenesis. Interestingly, HTLV-1 also encodes a protein called Hbz from the antisense strand of the proviral genome that counters many Tax functions in the infected cell, such as Tax-mediated viral transcription and NF-κB activation. However, Hbz also promotes cellular proliferation, inhibits apoptosis, and disrupts genomic integrity. In addition to viral proteins, there are other cellular factors such as MEF-2, superoxide-generating NAPDH oxidase 5-α (Nox5α), and PDLIM2 which have been shown to be critical for HTLV-1-mediated T-cell transformation. This review will highlight the important viral and cellular factors involved in HTLV-1 transformation and the available *in vitro* and *in vivo* tools used to study this complex process.

## Introduction

Human T-cell leukemia virus type 1 (HTLV-1) is a complex δ retrovirus within the *Orthoretrovirinae* subfamily. Isolated over 40 years ago from a patient with cutaneous T-cell lymphoma, HTLV-1 was the first discovered human retrovirus [1]. Currently, an estimated 5–10 million people worldwide are infected with HTLV-1, with pockets of endemic infection in Africa, South America, the Caribbean, Southwestern Japan, and the Pacific islands [2]. Large portions of the world lack epidemiological data regarding HTLV-1 infection; therefore, the actual number of HTLV-1-infected individuals is likely much higher. This bloodborne pathogen has the potential to induce adult T-cell leukemia (ATL), an extremely aggressive CD4^+^ T-cell malignancy [1,3], HTLV-1-associated myelopathy/tropical spastic paraparesis (HAM/TSP), a progressive neurodegenerative disease [4,5], and several other inflammatory diseases (uveitis, keratitis, dermatitis, and conjunctivitis) [6]. Approximately 5–10% of HTLV-1-infected patients will develop disease over the course of their lifetime. Unlike many other oncogenic retroviruses, HTLV-1 does not capture a proto-oncogene or induce proviral insertional mutagenesis. Instead, HTLV-1 is a trans-activating retrovirus and encodes accessory proteins that induce cellular transformation over an extended period of time. Therefore, in addition to lower disease penetrance, HTLV-1-mediated disease is unique due to its extensive clinical latency period upwards of several decades. This prolonged clinical latency period is heavily reliant on the transient expression of the viral regulatory protein, Tax. Although Tax and a subset of HTLV-1 accessory proteins have been directly implicated in the capacity of HTLV-1 to transform cells, the precise mechanisms of this process still remain largely unknown.

HTLV-1 has several routes of transmission, including breastfeeding, sexual intercourse, and exposure to infected blood products such as through blood transfusions or sharing of needles. Mother-to-child transmission through breastfeeding is typically the most common transmission route within endemic regions [7]. While most viruses are capable of infecting target cells through cell-free virions, transmission of HTLV-1 is primarily dependent on cell-to-cell transmission [8]. This phenomenon is partly due to the severely limited capacity of cell-free HTLV-1 virions within the blood to infect most cell types [9]. Interestingly, an *in vitro* study has suggested that transmission of HTLV-1 through a route such as breastfeeding requires transcytosis of free infectious HTLV-1 virions through the epithelial barrier [10]. These virions could subsequently infect dendritic cells (DCs), which are able to spread the HTLV-1 virions to healthy T cells [11]. Among the T-cell population, CD4^+^ T cells are the primary and preferential target cell of HTLV-1 infection, with CD8^+^ T cells constituting roughly 5% of the total infected cells [12,13]. During the acute stage of HTLV-1 infection, cell-to-cell transmission is prevalent resulting in efficient spread of the virus with limited immune detection. In addition to being hypothetically passaged to healthy T cells via infected antigen-presenting DCs, HTLV-1 virions can also be transmitted to adjacent cells through the following mechanisms: establishment of cellular conduits, formation of a virological synapse (VS), or through extracellular viral assemblies [14].

**Figure 1:**
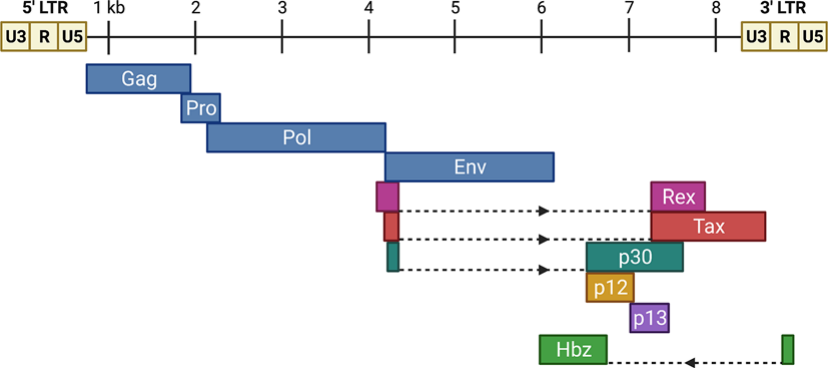
The HTLV-1 proviral genome The structural and enzymatic proteins (Gag, Pro, Pol, and Env), in addition to five viral accessory proteins (Tax, Rex, p12, p13, and p30), are encoded by sense transcripts derived from the 5′ LTR promoter. Hbz is the only viral accessory/regulatory protein encoded by an antisense transcript derived from a 3′ LTR promoter.

The HTLV-1 viral genome consists of a relatively small positive-sense RNA genome of approximately 9 kb in size. Two copies are packaged in the virus particle. Upon entry into a host cell, the ssRNA genome is reverse transcribed into dsDNA and integrated into the host genome. The integrated dsDNA form of the retroviral genome is termed the provirus. The viral genome is flanked by long terminal repeats (LTRs) at both the 5′ and 3′ ends. These direct repeats consist of three regions: the unique 3′ (U3), the repeated (R), and the unique 5′ (U5) regions. The LTRs also contain important elements necessary for viral transcription, polyadenylation, and integration. Like all retroviruses, HTLV-1 contains the standard structural and enzymatic genes (*gag*, *pro*, *pol*, and* env*) essential for viral replication (Figure 1). However, HTLV-1 is a complex retrovirus and also encodes accessory genes that contribute to the biology of the virus, of which a subset is critical to the pathogenesis. With the exception of one gene (*Hbz*), all the HTLV-1 genes are encoded from the sense strand of the proviral genome. One of the most critical accessory genes for HTLV-1 is *Tax*. Tax protein is required for transformation and functions as a transcription factor to enhance viral transcription from the promoter in the 5′ LTR [15–18]. Conversely, the Hbz protein, encoded from the antisense genome strand counteracts many Tax activities, including transcriptional activation of the 5′ LTR promoter [19–21]. Together, Tax and Hbz are essential for the ability of the virus to establish persistent infection and induce transformation of target T cells. This review will focus on both the viral factors (Tax, Hbz) and cellular players involved in HTLV-1-mediated cellular transformation, and the molecular tools and animal models currently available to study this dynamic process.

## Viral proteins relevant to HTLV-1-mediated transformation

Of all the HTLV-1 proteins, Tax is the most important for driving HTLV-1-mediated T-cell transformation and its transformation capacity has been well demonstrated experimentally. Previous studies have shown that Tax plays a major role in the processes of HTLV-1-mediated T-cell immortalization of primary human lymphocytes, colony formation in rodent fibroblasts, and tumorigenesis in a variety of transgenic (Tg) mouse models [22–25]. As a virally encoded oncoprotein, Tax has extensive involvement in a multitude of different pathways related to the immortalization, gene expression, and pathogenesis of HTLV-1. The multifunctionality of Tax throughout these respective processes is derived from its capability to directly interact with a wide array of proteins, including transcription factors, cell signaling proteins, cell cycle regulators, apoptotic proteins, and DNA damage response factors (Table 1). These interactions favor transformation and pathogenesis by modulating viral gene expression, deregulating the cell cycle, disrupting the apoptotic response, and reducing genomic stability.

**Table 1:** Interacting partners of Tax

Interacting partner/pathway	Downstream effect	References
CREB/AFT/p300	Transactivation of the 5′ LTR promoter and viral mRNA transcription	[28–31]
HDAC1	Prevents HDAC1’s interaction with the 5′ LTR, promoting viral transcription	[32]
SUV39H1	Recruits SUV39H1 to the promoter region, repressing viral transcription	[33]
DROSHA	Targets DROSHA for proteasomal degradation, increasing viral transcription	[36]
NEMO	Induces aberrant NF-κB activation	[38]
IκB protein	Targets IκB for proteasomal degradation, promoting NF-κB activation	[39]
p105	Targets p105 for proteasomal degradation, promoting NF-κB activation	[39]
NF-κB proteins	Promotes dimerizationof NF-κB proteins to enhance their transcriptional potential	[40]
TAB2	Stimulates the IKK complex and induces aberrant NF-κB activation	[41]
RFN8	Stimulates the IKK complex and induces aberrant NF-κB activation	[42]
PP2A	Inactivates PP2A, promoting NF-κB activation	[43]
A20	Inactivates A20, promoting NF-κB activation	[44]
TRAF6	Promotes the stability of MCL-1, preventing apoptosis induction	[50]
p53	Indirect inhibition deregulates the cell cycle and promotes T-cell immortalization	[55]
Ras proteins/mTOR pathway	Tax-mediated activation promotes accelerated cell proliferation	[56,57]
MDAC1	Sequesters MDAC1, causing sustained DNA damage and increased mutations	[61,62]
USP10	Causes induction of reactive oxygen species	[66]

Abbreviations: AFT, activating transcription factor; A20, ubiquitin-editing enzyme A20; CREB, cyclic AMP response element-binding protein; DROSHA, Drosha ribonuclease III; HDAC1, histone deacetylase 1; IKK, IκB kinase; MCL-1, myeloid cell factor-1; MDAC1, mediator of DNA damage checkpoint 1; NEMO, NF-κB essential modulator; PP2A, protein phosphatase 2A; RFN8, ring finger protein 8; TAB2, TAK1-binding protein 2; TRAF6, TNF receptor-associated factor 6; USP10, ubiquitin-specific peptidase 10.

One of the key roles of Tax is transactivation of the promoter located in the 5′ LTR. Tax-mediated viral transcription drives synthesis of all plus strand gene products including the structural and enzymatic viral proteins, in addition to regulating its own expression. Tax mediates viral transcription through three discontinuous G/C-rich 21-base pair repeats, known as Tax responsive elements (TREs), located within the U3 region of the 5′ LTR [26,27]. These G/C-rich regions are directly adjacent to cyclic AMP response elements (CREs) and influence transcription when bound to transcription factors such as CRE-binding protein (CREB)/activating transcription factor (ATF) [28–30]. Tax does not directly bind DNA, but instead recruits transcription factors (CREB) to the CRE. This ultimately leads to a nucleoprotein complex that strongly favors recruitment of additional transcriptional co-activators and histone acetylases, such as CREB-binding protein (CBP) and p300. This multiprotein complex consisting of Tax, CREB/ATF transcription factors, and CRE/p300 histone acetylases leads to potent transactivation of the 5′ LTR promoter and viral mRNA transcription [31]. Tax also regulates HTLV-1 gene transcription through binding to repressive epigenetic proteins such as histone deacetylase 1 (HDAC1) and the histone lysine methyltransferase suppressor of variegation 3-9 homolog 1 (SUV39H1) [32,33]. Tax has the ability to either promote viral transcription by directly preventing HDAC1 interaction with the viral promoter or repress viral transcription through the active recruitment of SUV39H1 to the promoter region. Another mechanism of Tax-mediated regulation of viral replication and gene transcription is the dysregulation of cellular miRNA expression, which has been frequently identified across HTLV-1-infected T-cell lines and ATL patient samples [34,35]. For the purposes of increasing viral transcription, Tax can disrupt miRNA machinery by targeting Drosha ribonuclease III (DROSHA) for proteasomal degradation [36].

**Figure 2:**
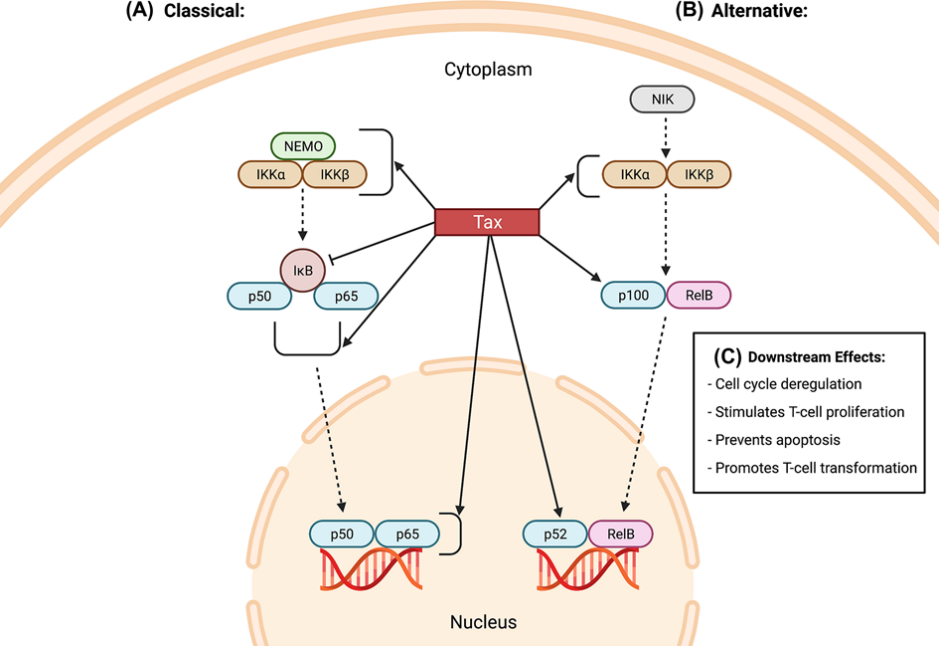
Tax-mediated activation of the NF-κB pathway HTLV-1-infected cells utilize Tax to stimulate both the classical (**A**) and alternative (**B**) NF-κB pathways. (A) Tax interacts with NEMO to facilitate recruitment of the IKK subunits α and β, leading to activation of the IKK complex. Tax can also interact with these IKK subunits directly to promote their dimerization. The activated IKK complex then phosphorylates IκB, subsequently releasing p50/p65 and permitting their translocalization to the nucleus. Tax can also bind to p50/p65 subunits in the nucleus to induce dimer formation and recruit co-activators such as CBP/p300. (B) The alternative pathway depends on phosphorylation of the IKK complex by NIK. Once activated, the IKK complex then phosphorylates p100/RelB, stimulating proteasomal processing of p100 to p52. Tax accelerates this process by recruiting IKK subunit α to p100, thereby allowing p52/RelB to translocate to the nucleus. Similar to the classical pathway, Tax may additionally promote the dimerization and DNA binding activity of nuclear p52. (**C**) Tax-mediated activation of these respective NF-κB pathways leads to several downstream cellular effects, including deregulation of the cell cycle, stimulation of T-cell proliferation, and increased expression of anti-apoptotic proteins, ultimately promoting viral transformation of HTLV-1-infected cells.

In addition to regulating viral gene expression, Tax also has a strong influence over several cell signaling pathways such as NF-κB (Figure 2). This activity of Tax is particularly relevant given that the NF-κB transcription factor family plays several critical roles in the processes of apoptosis, cell proliferation, oncogenesis, and immune response development. Activation and nuclear translocation of cytoplasmic NF-κB proteins is dependent on the phosphorylation and proteasomal degradation of IκB proteins, which are commonly bound to NF-κB dimers and thus masking their nuclear localization signal [37]. Tax induces aberrant NF-κB activation by interacting with the NF-κB essential modulator (NEMO) on the IκB kinase (IKK) complex, subsequently promoting this kinase to actively phosphorylate IκB proteins bound to NF-κB dimers [38]. Alternatively, Tax can also directly bind to IκB proteins, such as IκBα and p105, to disrupt their NF-κB binding interactions and target them for proteasomal degradation [39]. Tax also has the capacity to enhance the transcriptional potential of NF-κB proteins by actively promoting their homo- or hetero-dimerization through direct interactions with their respective Rel homology domains [40]. In order to ensure persistent activation of NF-κB within the cell, Tax is able to interact with a wide subset of proteins involved in the regulation of NF-κB. For example, Tax can interact with TAK1-binding protein 2 (TAB2) and Ring Finger Protein 8 (RFN8), two unique proteins involved in the downstream stimulation of the IKK complex [41,42]. Tax also binds to and inactivates negative regulators of the NF-κB signaling pathway, such as the serine/threonine protein phosphatase PP2A and the ubiquitin-editing enzyme A20 [43,44]. In summation, Tax possesses a substantial array of different mechanisms to trigger and maintain the persistent activation of NF-κB signaling within HTLV-1-infected cells. Given that chronic activation of NF-κB has been shown to be critical for the immortalization and transformation of Tax-expressing cells, HTLV-1 transformed cell lines, and ATL patient samples alike, the NF-κB pathway represents a key mechanism for Tax to influence the transformation of HTLV-1-infected cells [45,46].

Persistent activation of NF-κB results in stimulation of growth factors, cytokines, and proto-oncogenes—all of which influence the host cell cycle. NF-κB signaling also has the ability to activate expression of anti-apoptotic proteins. Not surprisingly, Tax-mediated activation of NF-κB has been reported to enhance expression of the anti-apoptotic proteins X-linked inhibitor of apoptosis (XIAP), survivin, and Bcl-xL [47–49]. Tax can also protect HTLV-1-infected cells from apoptosis through other mechanisms, such as interactions with TRAF6 to promote the stability of myeloid cell factor-1 (MCL-1), an anti-apoptotic protein of the BCL-2 family [50]. Although Tax typically functions to increase the expression of anti-apoptotic proteins, studies have demonstrated that overexpression of Tax may result in the up-regulation of pro-apoptotic proteins [51]. Tax overexpression in the CD4^+^ T-cell line Jurkat leads to enhanced expression of the pro-apoptotic ligands CD95L, TNF-α, and TNF-related apoptosis inducing ligand (TRAIL), ultimately leading to widespread cell death [52–54]. In addition to inhibiting apoptosis, Tax also functions to regulate and stimulate T-cell proliferation. Tax-mediated inhibition of the tumor suppressor p53 is one of the most prominent methods utilized to deregulate the cell cycle and promote HTLV-1-mediated T-cell immortalization. Tax inhibits p53 indirectly through activation of either NF-κB or CREB cell signaling pathways [55]. In addition, Tax promotes accelerated cell proliferation through the selective activation of Ras proteins and the mTOR signaling pathway [56,57]. Tax can also increase cell proliferation through NF-κB-mediated induction of oncogenesis-related cellular miRNAs, miR-146a and miR-155 [58,59]. By enhancing the capacity of the cell to proliferate while simultaneously up-regulating the expression of anti-apoptotic proteins, Tax is effectively able to drive the immortalization and transformation process in HTLV-1-infected T cells.

Through modulation of several proteins critical for proper cell cycle regulation, Tax can also promote the transformation of HTLV-1-infected T cells by preventing the recognition and repair of DNA damage. The ataxia-telangiectasia mutated (ATM) and ATM and Rad3-related (ATR) kinases exert cell cycle delay in response to compromised genomic integrity such as DNA damage or stalled replication forks [60]. Tax prevents the activation of ATM through sequestration of mediator of DNA damage checkpoint 1 (MDAC1), subsequently resulting in sustained DNA damage and a higher genomic mutation frequency [61,62]. Through its interactions with the CREB/ATF transcription factors, Tax can also decrease cyclin A promoter expression. Given that cyclin A kinase activity is vital for maintaining the proper levels of DNA replication during S phase, Tax-mediated suppression of this promoter greatly enhances the rate of genetic instability and DNA damage [63,64]. In addition to blocking DNA repair mechanisms across several phases of the cell cycle, Tax also has the ability to directly induce DNA damage through the induction of reactive oxygen species (ROS) intermediates and nitric oxide (NO). Previous studies found that primary human CD4^+^ T cells transduced with various Tax-expressing retroviral vectors produced ROS as a direct consequence of Tax expression, subsequently causing DNA damage and the initiation of cell senescence [65]. The interaction between Tax and the deubiquitinase USP10 is one of the molecular mechanisms that leads to Tax-mediated induction of ROS [66]. The ability of Tax to induce both DNA damage and prevent genomic repair mechanisms during the cell cycle enhances the oncogenic and transformative properties of Tax.

Although the expression of Tax alone is sufficient to promote tumorigenesis in Tg mouse models *in vivo*, Tax by itself is not able to efficiently transform primary human cells *in vitro* [24,67,68]. Therefore, the transformation of HTLV-1-infected T cells is dependent on other viral proteins in addition to Tax. A promoter on the antisense strand in the 3′ LTR of the proviral genome drives expression of Hbz, the only viral protein derived from an antisense transcript in HTLV-1. A critical function of Hbz is to inhibit Tax-mediated activation of viral transcription within the nucleus of infected cells [69]. Hbz interacts with essential Tax transcription factors (ATF-1, CREB, JunB, JunD, and c-Jun) via its basic leucine zipper domain and sequesters them away from DNA, thus inhibiting Tax-mediated transcription [70]. In addition to suppressing activation of the 5′ LTR promoter, Hbz also has the capacity to antagonize other roles of Tax, such as stimulation of the classical NF-κB pathway. Through the antagonization and degradation of the NF-κB component p65, Hbz is able to suppress Tax-driven activation of the classical NF-κB pathway, which consequently results in the attenuation of the cellular senescence response [71]. Decreasing the activity of Tax is hypothesized to serve an important role in the survival of HTLV-1-infected cells and the controlled proliferation and clonal expansion of HTLV-1-infected CD4^+^ T cells is reliant on the Hbz-mediated down-regulation of Tax and NF-κB expression. Given the pathogenic and transformative properties of Tax, Hbz-mediated inhibition of this viral transcriptional activator additionally functions as a means to regulate the transformation process. However, loss of Hbz does not affect HTLV-1-mediated T-cell immortalization in cell culture [72]. Interestingly, the absence of Hbz does affect efficient viral infectivity and persistence in a rabbit model of infection [72]. These studies demonstrate that Hbz is critical in the efficient establishment and maintenance of chronic viral infections.

Hbz can also interact with the AP1 superfamily proteins ATF3 and JunD [73–75]. ATF3 is a transcription factor and also activates the tumor suppressor p53. Hbz interferes with the p53-enhancing function of ATF3 and enhances JunD transcriptional activity to promote proliferation of T cells [74,76,77]. Somewhat surprisingly, recent advances in the field have shown that *hbz* mRNA is also able to support cell proliferation, although the precise mechanism(s) remain unclear [78]. Like Tax, Hbz also has the ability to promote genetic instability, a significant factor driving HTLV-1-mediated oncogenesis. Hbz has been previously reported to increase the expression of miR-17 and miR-21, two unique oncogenic miRNAs that down-regulate the OBFC2A DNA-damage factor, subsequently leading to a higher propensity for DNA damage [79]. Additional *in vitro* studies with murine cell lines have demonstrated that the independent expression of Hbz significantly promotes soft agar cell proliferation and colony formation [76]. Altogether, Hbz functions as a key viral protein for both the regulation and promotion of HTLV-1-mediated T-cell transformation. Similar to Tax, Hbz is also able to inhibit apoptosis of infected cells. Bim, a pro-apoptotic gene, is suppressed by Hbz expression, while knockdown of Hbz increases Bim expression [80]. Further investigation of the mechanism behind the Hbz/Bim relationship found that Hbz disrupts the localization and function of FoxO3a, a critical activator of Bim and also Fas ligand (FasL).

Similar to Hbz, many of the remaining viral accessory proteins of HTLV-1 also play a key role in regulating Tax activity and the transformation potential of virally infected T cells [81]. The viral protein p30 is characterized as a negative post-transcriptional regulator of viral gene expression and Tax activity; p30 selectively binds to *tax/rex* mRNA transcripts and prevents their export from the nucleus [82]. Furthermore, p30 has been shown to prevent Tax-mediated transactivation of the 5′LTR promoter by interacting with CBP/p300 [83]. Previous studies have additionally indicated that p30 has the capacity to dampen Tax-mediated NF-κB activation by destabilizing the p65 subunit [84]. Despite the ability of p30 to antagonize Tax, this viral protein can also promote T-cell transformation. Previous *in vitro* studies using immortalized human fibroblasts demonstrated that p30 can enhance viral transformation when coexpressed with Myc [85]. An additional viral accessory gene called p13 also disrupts the interaction between Tax and CBP/p300, resulting in the down-regulation of viral transcription [86]. p13 has also been implicated in elevated levels of intracellular ROS, subsequently enhancing the potential for DNA damage and viral transformation [87,88]. In contrast with p30 and p13, expression of the viral accessory protein p12 is typically not involved with suppression of Tax activity. Contrary, this viral protein enhances the potential for CD4^+^ T-cell proliferation through activation of nuclear factor of activated T cells (NFAT) and signal transducers and activators of transcription-5 (STAT-5) signaling pathways [89–91]. Several studies have also shown p12 is able to enhance viral infectivity and immune evasion [91–93].

Tax is the major driver of 5′ viral transcription and thus has tight control over its own expression. In addition, Tax regulates cellular epigenetic proteins and miRNA machinery which influences the immortalization process. Several viral accessory proteins, including Hbz, function to regulate Tax activity, viral gene expression, and T-cell transformation through a variety of different mechanisms.

## Cellular factors relevant to HTLV-1-mediated transformation

Transformation of HTLV-1-infected T cells is mediated by a wide variety of both viral and cellular proteins. Although viral proteins such as Tax and Hbz play an important role in T-cell transformation, there are several additional cellular factors that contribute to this process (Table 2). These cellular factors become increasingly relevant later during viral infection, as Tax expression is typically silenced in the majority of ATL patient samples [94]. Furthermore, a long clinical latency period suggests there are additional genetic and/or epigenetic events which occur to induce oncogenesis [95]. An integrated molecular study with whole-genome, exome, transcriptome, and targeted sequencing of 426 ATL cases found that despite the absence of Tax, a number of new somatic alterations were identified, and these alterations were found in T-cell receptor (TCR) and NF-κB signaling [96]. These pathways have a significant overlap with the Tax interactome despite the absence of Tax in these patient samples.

**Table 2:** Cellular factors involved in HTLV-1 transformation

Cellular factor	Transformation target/effect	References
Nox5α	Up-regulation helps maintain the survival, proliferation, migration, and oncogenic potential of HTLV-1-transformed cells	[93]
MEF-2	Colocalizes with Tax to stabilize its interaction with CREB, causing increased viral transcription	[95]
PDLIM2	Targets Tax for proteasomal degradation, decreasing the transformation capactiy of infected cells	[96]
PTHrP	Up-regulation maintains the transformation of infected cells	[98,99]
PRMT5	Up-regulation promotes the survival and transformation of infected cells	[100]
miR-93	Targets p21, MICB, and TP53INP1; increases the oncogenic potential and immune evasion of infected cells	[102–104]
miR-130b	Targets TP53INP1; maintains the survival of infected cells	[104]
miR-150	Functions as a tumor suppressor by repressing the proliferation of transformed cells	[105]

Abbreviations: MEF-2, myocyte enhancer factor-2; MICB, major histocompatibility complex class I chain-related B; Nox5α, superoxide-generating NAPDH oxidase 5-α; PDLIM2, PDZ and LIM domain 2; PRMT5, protein arginine methyltransferase 5; PTHrP, parathyroid hormone-related protein; TP53INP1, tumor protein 53-induced nuclear protein 1.

Notably, a study by Shigemura et al*.* revealed that superoxide-generating NAPDH oxidase 5-α (Nox5α) is a cellular protein that is required for the maintenance of HTLV-1-transformed T cells [97]. This study demonstrated that Nox5α is up-regulated in both ATL patient samples and HTLV-1 transformed cell lines such as Hut-102, MT1, MT2, and MT4. Furthermore, siRNA-mediated silencing of Nox5α in MT1 and MT2 cell lines resulted in reduced T-cell proliferation and migration, in addition to increased apoptosis. Through the use of NOG mice transplanted with siRNA-transfected MT2 cells, the present study also illustrated that *in vivo* down-regulation of Nox5α led to an observable decrease in the tumor growth rate of HTLV-1 tumor transplant mice. In summation, expression of Nox5α is critical for maintaining the survival, proliferation, migration, and oncogenic potential of HTLV-1 transformed cells, and therefore serves as a key cellular factor in promoting the transformation phenotype of HTLV-1-infected T cells [97].

Several cellular proteins have been shown to be involved in HTLV-1-mediated T-cell transformation by interfering with the activity and expression of Tax. A study by Jain et al*.* demonstrated that myocyte enhancer factor-2 (MEF-2), a transcription factor for Interleukin 2 (IL-2) produced during peripheral T-cell activation, is substantially up-regulated within HTLV-1-infected cells and ATL patient samples [98,99]. Through shRNA-mediated knockdown within an *in vitro* co-culture system, MEF-2 was found to be essential for the transformation of CD4^+^ T cells by HTLV-1. Furthermore, direct inhibition of MEF-2 activity and expression resulted in decreased viral replication and transactivation of the 5′ LTR promoter, which was attributed to the propensity of MEF-2 to colocalize with Tax and stabilize its interaction with CREB. Therefore, MEF-2 is not only required for viral transformation but is also critical for the ability of Tax to efficiently promote viral gene expression from the 5′ LTR promoter [99]. In contrast with MEF-2, the ubiquitin E3 ligase, PDLIM2, functions to repress the transformation of HTLV-1-infected T cells by directly targeting Tax for proteasomal degradation at the nuclear matrix [100]. This role of PDLIM2 in HTLV-1 transformation was revealed in a study by Yan et al*.*, which characterized PDLIM2 mRNA and protein to be highly expressed in HTLV-1-negative Jurkat cells and significantly down-regulated in HTLV-1-transformed T-cell lines such as C8166, MT4, Hut-102, and SLB-1. Co-expression of PDLIM2 and Tax in HTLV-1-transformed cell lines led to a dose-dependent decrease in Tax-mediated NF-κB activation and viral transcription, while PDLIM2 knockout reversed this effect. In addition, artificial up-regulation of PDLIM2 in Tax-expressing Rat-1 fibroblasts suppressed the growth of these cells in soft agar, suggesting that PDLIM2 plays an important role in reducing the capacity of HTLV-1-infected cells to transform and proliferate *in vitro* [100]. Expression of PDLIM2 also had a significant impact *in vivo* since severe combined immunodeficiency (SCID) mice transplanted with C8166 and MT4 cells exhibited no tumorigenesis when co-expressed with both PDLIM2 and Tax [100]. Regulation of PDLIM2 has been strongly suggested to be mediated by the DNMT1 and DNMT3b methyltransferases, which are both significantly up-regulated in a multitude of different HTLV-1-transformed T-cell lines [101]. Altogether, MEF-2 and PDLIM2 are cellular proteins that function to either enhance or repress the transforming capacity of HTLV-1-infected cells by modulating the activity of Tax.

Another notable transforming factor for HTLV-1 is parathyroid hormone-related protein (PTHrP), which is constitutively expressed in HTLV-1 carriers and highly up-regulated during HTLV-1-mediated transformation of T cells [102,103]. Compared with an established HTLV-1 cell line such as MT2, the promoter of PTHrP was preferentially activated within recently immortalized HTLV-1 cell lines. In addition, the transient expression of Tax was not correlated with the expression pattern of PTHrP, indicating that this cellular protein likely plays a role in T-cell transformation independent of Tax activity [102]. Similar to PTHrP, expression of protein arginine methyltransferase 5 (PRMT5) has also been implicated in HTLV-1-mediated T-cell transformation. PRMT5 was up-regulated in both HTLV-1 transformed cell lines, ATL-derived cell lines, and during HTLV-1-mediated T-cell transformation *in vitro* [104]. This protein is also essential for the survival of HTLV-1 transformed cells since enzymatic inhibition of PRMT5 activity results in significant dose-dependent toxicity in HTLV-1-transformed cell lines such as SLB-1 and Hut-102 [104]. Although the precise mechanisms of how PTHrP and PRMT5 directly contribute to the viral transformation process have yet to be fully elucidated, the elevated expression levels of these cellular factors within immortalized cell lines strongly suggest that they play a unique role.

miRNAs have also been shown to play many diverse and notable roles in the process of HTLV-1-mediated T-cell transformation. A review by Ruggero et al*.* outlines a large array of different miRNAs that have been reported to be up-regulated in HTLV-1-transformed T-cell lines and ATL-derived patient cell lines [105]. As described previously in this review, several miRNAs are completely dependent on the expression of specific viral proteins to promote cellular transformation, such as miR-155 with Tax and miR-21 with Hbz [58,79]. However, not all cellular miRNAs which affect HTLV-1-mediated transformation are controlled or regulated by viral gene products. For example, up-regulation of miR-93 in HTLV-1-transformed and ATL-derived cell lines has been linked to the expression of the E2F1 transcription factor. The oncogenic potential of miR-93 is derived from its tendency to target key cell cycle regulators such as p21 [106]. miR-93 can also aid in viral immune evasion by significantly down-regulating the expression of histocompatibility complex class I chain-related B (MICB), which serves as a major ligand for natural killer (NK) cells and CD8^+^ T cells [107]. In addition, miR-93 has been shown to function alongside miR-130b to specifically target tumor protein 53-induced nuclear protein 1 (TP53INP1), a tumor suppressor protein that exhibits relatively minimal expression in HTLV-1-transformed and ATL-derived cell lines. Antagonization of miR-93 and miR-130b in the HTLV-1-transformed cell line MT4 was reported to substantially reduce cell viability, demonstrating the importance of these miRNAs for the survival of HTLV-1-transformed cells [108]. However, not all up-regulated miRNAs in ATL patient lines have been reported to enhance the transformed phenotype. Shown to be up-regulated in ATL patient samples but down-regulated in HTLV-1-transformed cell lines, miR-150 has been reported to repress the proliferation of transformed cells by functioning as a tumor suppressor [109]. Cellular factors and miRNAs have a wide variety of diverse functions that can serve to either promote or suppress HTLV-1-mediated T-cell transformation independent of Tax or Hbz.

## *In vitro* and *in vivo* models useful for transformation studies

**Figure 3:**
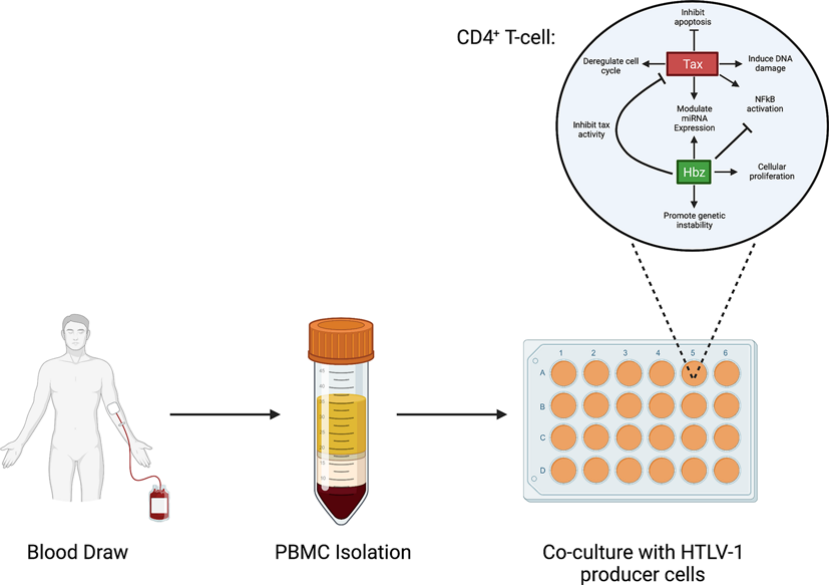
HTLV-1 co-culture immortalization assay Human PBMCs are obtained from a healthy blood donor and immediately isolated through Ficoll density gradient separation. Freshly isolated PBMCs are subsequently co-cultured with lethally irradiated virus producer cells where CD4^+^ T cells will become infected with HTLV-1. Within the infected cell, Tax and Hbz viral proteins are expressed. Over a period of several weeks, these viral proteins play a prominent role in HTLV-1-mediated T-cell transformation by influencing a multitude of different cellular functions, as depicted above.

HTLV-1 is able to transform primary CD4^+^ T cells *in vitro* using a co-culture immortalization assay [110]. The use of this assay requires freshly isolated peripheral blood mononuclear cells (PBMCs) from a healthy donor co-cultivated with an HTLV-1 producer cell line (Figure 3). A viral producer cell line is essential as cell-free infection by HTLV-1 is extremely inefficient and successful infection requires the co-cultivation of infected cells with naïve target cells. The HTLV-1 producer cell line is typically subjected to lethal irradiation immediately prior to co-culture. This ensures that virions are successfully transmitted to target cells and producer cells are eliminated from the long-term co-culture. Initiation of transformation becomes apparent within 5-6 weeks following co-culture as detected by expansion of CD4^+^ T cells from the PBMCs mixed cell population. This type of assay allows for not only the study of early viral infection events, but also the ability of HTLV-1 to immortalize a target cell. Cell viability, cell number, and the number of transformed wells can be measured at weekly time points to assess the immortalization process. This type of assay has been useful for studying the function and biochemistry of individual viral proteins and protein modifications and their role in transformation [72,111–113]. Not only is this assay cost effective, but it is readily available for many labs with access to cell culture resources. The limitation of this type of study is the absence of a functional immune response and therefore selective pressure. Indeed, viral genes which have been shown to be essential for viral persistence and establishment of infection *in vivo*, such as Hbz, are not required for transformation *in vitro* [72,111].

Mouse models have provided a useful tool for studying cellular and viral factors required for development and maintenance of HTLV-1-mediated transformation. Some of the earliest mouse studies within the HTLV-1 field involved the use of Tax Tg mice [114–116]. These initial studies were useful for understanding the role of Tax and Tax-mediated disturbance of normal lymphocyte function. However, these early Tax Tg mouse models had tissue-specific expression patterns uncharacteristic of HTLV-1-infected patients. In an effort to restrict Tax expression to the lymphoid cell compartment, subsequent studies used alternative promoters to drive Tax expression. Tax driven by the granzyme B (Gzb) promoter was able to target expression to the mature T-cell compartment [24]. The Gzb-Tax Tg mice developed large granular lymphocytic leukemia/lymphoma and also spontaneously developed hypercalcemia, high frequency osteolytic bone metastases, and enhanced osteoclast activity—all symptoms frequently found in ATL patients. Subsequent studies have used other promoters (CD3-ϵ promoter–enhancer sequence, Lck proximal promoter) to drive Tax expression or coupled Tax Tg with LTR-luc Tg mice to monitor inflammation and lymphoma development using non-invasive bioluminescent imaging [25,68,117–119]. Shortly after the discovery of Hbz, Tg mice expressing Hbz in CD4^+^ T cells were developed [120]. Hbz Tg mice developed T-cell lymphomas and system inflammation along with elevated levels of CD4^+^ Foxp3^+^ T(reg) cells and effector/memory CD4^+^ T cells. Tg mice have been especially useful for establishing the role of Tax and Hbz in transformation and oncogenesis. These mice also provide a valuable tool to test various future treatments, particularly those targeted at Tax or Hbz, and to investigate additional cellular factors involved in transformation and disease development.

In the early nineties, SCID mice were used to successfully model the proliferative and tumorigenic potential of ATL cells [121]. Of note, these mice were also treated with an anti-asialo GM-1 antibody to abolish NK cell activity and enhance the growth of lymphoma. Subsequent studies found that tumors developed in SCID mice injected with peripheral blood lymphocytes (PBLs) from ATL patients, but not with PBLs generated by *in vitro* co-culture immortalization assays [122]. Taken together, these data suggest that the genetic environment plays an important role in tumorigenesis and that cells from a non-leukemic origin have not gathered the essential genetic alterations needed to reach the full tumorigenic potential *in vivo*. NOD/SCID γ c (null), or NOG mice, have immunological dysfunctions of T, B, and NK cells. Successful transplantation of various HTLV-1-transformed cell lines (SLB-1, Hut-102, and TL-om1) into these mice has been beneficial for examining viral-induced cell proliferation and tumorigenesis [123]. For example, SLB-1 cells with shRNA-mediated knockdown of Hbz had significantly reduced tumor burden and cell proliferation in NOG mice compared with wildtype SLB-1 cells, thus demonstrating the proliferative capacity of Hbz *in vivo* [124]. Like Tg mice, NOG mice represent an *in vivo* model for studying HTLV-1-mediated transformation and the development of novel drugs and targets to reduce disease burden.

In efforts to develop an improved model of HTLV-1 infection and disease progression, ‘humanized immune system’ (HIS) mouse models were developed. NSG mice, or NOD-SCID/γ(null) (NOD.Cg-*Prkdc^scid^Il2rg^tm1Wjl^* /SzJ) mice, lack mature T, B, and NK cells, are deficient in multiple cytokine signaling pathways, and have many defects in innate immunity. HIS mice are created by injecting human umbilical-cord stem cells (CD34^+^) into the livers of immunodeficient neonatal NSG mice, resulting in the development of human lymphocytes that appear phenotypically normal but cannot mount an adaptive immune response [125,126]. HIS mice inoculated with HTLV-1 consistently reproduce the three key stages of HTLV-1-induced tumorigenesis in a very compact time frame (approximately 4–5 weeks): (1) persistent infection, (2) chronic proliferation of CD4^+^ T cells, and (3) development of lymphoproliferative disease. A recent study using these mice found that the envelope protein of HTLV-1 is important to induce CD4^+^ T-cell proliferation (vs. CD8^+^ T-cell proliferation), suggesting that T-cell transformation *in vivo* may also be influenced by the Env protein of the virus [125]. The ability to incite disease in this model allows for the study of T-cell transformation and tumorigenesis, as well as interaction of tumor cells with the microenvironment. Specific hurdles in the use of HIS mice are the cost associated with generating the mice, the maintenance, and care, but also the lack of a functional immune system. Recently, a novel HTLV-1-infected humanized mouse model was generated through intra-bone marrow injection of human CD133^+^ stem cells into NOG mice (IBMI-huNOG mice) [127]. After HTLV-1 infection, an increase in CD4^+^ T cells was observed in the periphery and atypical lymphocytes which represent ATL-specific flower cells were observed after 4–5 months. Proliferation and proviral load were also detected in CD4^+^ T cells. Importantly, the authors also report the presence of an HTLV-1-specific adaptive immune response in these animals. While technically challenging to create, this model will undoubtedly be useful for investigating *in vivo* mechanisms of transformation and disease development, as well as drug and vaccine candidates.

Recently, a Tg *Drosophila melanogaster* model expressing Tax in the compound eye and plasmatocytes (a leukocyte-like cell) has been described [128]. Tax expression induces a disruption of crystalline array of the ommatidia and an increase in plasmatocyte proliferation, demonstrating that Tax has transforming potential in the fly model. Not surprisingly, the induction of this eye phenotype is primarily dependent on the *Drosophila* homolog of IKKγ/NEMO. Conversely, an Hbz transgenic *Drosophila* model neither induces transformation nor NF-κB activation *in vivo* [129]. Instead, Hbz overexpression prevents Tax-mediated NF-κB activation and rescues the Tax-induced transformation phenotype. *Drosophila* models possess several advantages including fast generation time, high numbers of progeny, and several genetic screens (near complete collection of mutants and RNAi lines). In addition, several key signaling pathways are highly conserved between mammals and flies. However, while initial findings support this model system, any results from this model still require further validation in mammalian systems.

A key to the HTLV-1 transformation process is establishment of viral infection and viral persistence. HTLV-1 infection of rabbits mimics early infection in humans. New Zealand white rabbits inoculated with HTLV-1 become persistently infected [72,113,130]. The early rabbit humoral antibody responses against Gag and Env mimic asymptomatic early viral infection in humans. While these animals do not develop disease, they do recapitulate viral persistence (i.e., long-term viral latency) and enable the study of early viral infection events. An obvious limitation with the rabbit model is that it is not a disease model. Nevertheless, this animal model is invaluable for measuring early viral infection events and persistence in the presence of a functional immune system—factors which enable viral transformation.

## Conclusions

HTLV-1 is a transactivating retrovirus that has the potential to induce ATL and/or various inflammatory diseases following an extensive clinical latency period of up to several decades. Given the nature of ATL to be an extremely aggressive and chemotherapy resistant CD4^+^ T-cell malignancy that is often fatal, there is an urgent need to understand the viral and cellular mechanisms that regulate or influence HTLV-1-mediated T-cell transformation. Serving as the primary driving force for viral transformation, the HTLV-1-encoded Tax oncoprotein is critical for viral gene expression, immortalization, and pathogenesis. Through its ability to directly interact with a multitude of different cellular proteins and pathways, Tax can transform infected T cells by modulating viral gene expression at the 5′ LTR promoter, deregulating the cell cycle, disrupting the apoptotic response, and decreasing genomic stability. Many of these processes are heavily dependent on Tax-mediated activation of NF-κB. Antagonization of Tax-driven NF-κB induction and viral transcription is mediated by the viral protein Hbz. Derived from the anti-sense strand of the proviral genome, Hbz is required for the controlled proliferation and clonal expansion of HTLV-1-infected CD4^+^ T cells. Hbz can also promote the transformation of infected T cells by preventing apoptosis and reducing genomic stability. Tax and Hbz are not the only viral accessory proteins that influence the transformation of HTLV-1, as p12, p13, and p30 have individually been shown to play unique roles that contribute to this dynamic process. In addition to viral proteins encoded by HTLV-1, a multitude of cellular factors have also been illustrated to either promote (Nox5α and MEF-2) or repress (PDLIM2) the transformation of HTLV-1-infected cells.

Molecular tools such as co-culture immortalization assays and *in vivo* models including rabbits, mice, and Drosophila have been critical in our understanding of this oncogenic virus. The role of Tax and Hbz in the transformation process is undisputed, however several unknowns remain: (1) How important are other viral proteins in the transformation process? (2) What is the timing and expression level of Tax and Hbz required for efficient transformation? (3) Which cellular factors are critical for regulating Tax/Hbz? and (4) Which cellular factors are required to push an immortalized cell to oncogenesis/a diseased state? Since the discovery of HTLV-1 over 40 years ago, there has been considerable progress made towards understanding the viral transformation process. As the molecular tools and models improve, we will undoubtedly continue to refine our understanding of the dynamic HTLV-1-mediated cellular transformation process.

## Abbreviations

ATF: activating transcription factor
ATL: adult T-cell leukemia/lymphoma
ATM: ataxia-telangiectasia mutated
CBP/CREB: CREB-binding protein
CRE: cyclic AMP response element
HDAC1: histone deacetylase 1
HIS: humanized immune system
IKK: IκB kinase
LTR: long terminal repeat
NEMO: NF-κB essential modulator
NK: natural killer
NO: nitric oxide
Nox5α: NAPDH oxidase 5-α
PBL: peripheral blood lymphocyte
PBMC: peripheral blood mononuclear cell
PTHrP: parathyroid hormone-related protein
ROS: reactive oxygen species
SCID: severe combined immunodeficiency
SUV39H1: suppressor of variegation 3-9 homolog 1
Tg: transgenic

## References

[1] Poiesz B.J., Ruscetti F.W., Gazdar A.F., Bunn P.A., Minna J.D. and Gallo R.C. (1980) Detection and isolation of type C retrovirus particles from fresh and cultured lymphocytes of a patient with cutaneous T-cell lymphoma. Proc. Natl. Acad. Sci. U.S.A. 77, 7415–7419 10.1073/pnas.77.12.7415 6261256 PMC350514

[2] Gessain A. and Cassar O. (2012) Epidemiological aspects and world distribution of HTLV-1 infection. Front. Microbiol. 3, 388 10.3389/fmicb.2012.00388 23162541 PMC3498738

[3] Yoshida M., Miyoshi I. and Hinuma Y. (1982) Isolation and characterization of retrovirus from cell lines of human adult T-cell leukemia and its implication in the disease. Proc. Natl. Acad. Sci. U.S.A. 79, 2031–2035 10.1073/pnas.79.6.2031 6979048 PMC346116

[4] Gessain A., Barin F., Vernant J.C., Gout O., Maurs L., Calender A. et al. (1985) Antibodies to human T-lymphotropic virus type-I in patients with tropical spastic paraparesis. Lancet 2, 407–410 10.1016/S0140-6736(85)92734-5 2863442

[5] Osame M., Usuku K., Izumo S., Ijichi N., Amitani H., Igata A. et al. (1986) HTLV-I associated myelopathy, a new clinical entity. Lancet 1, 1031–1032 10.1016/S0140-6736(86)91298-5 2871307

[6] Martin F., Taylor G.P. and Jacobson S. (2014) Inflammatory manifestations of HTLV-1 and their therapeutic options. Expert Rev. Clin. Immunol. 10, 1531–1546 10.1586/1744666X.2014.966690 25340428

[7] Proietti F.A., Carneiro-Proietti A.B., Catalan-Soares B.C. and Murphy E.L. (2005) Global epidemiology of HTLV-I infection and associated diseases. Oncogene 24, 6058–6068 10.1038/sj.onc.1208968 16155612

[8] Pique C. and Jones K.S. (2012) Pathways of cell-cell transmission of HTLV-1. Front. Microbiol. 3, 378 10.3389/fmicb.2012.00378 23109932 PMC3479854

[9] Fan N., Gavalchin J., Paul B., Wells K.H., Lane M.J. and Poiesz B.J. (1992) Infection of peripheral blood mononuclear cells and cell lines by cell-free human T-cell lymphoma/leukemia virus type I. J. Clin. Microbiol. 30, 905–910 10.1128/jcm.30.4.905-910.1992 1572977 PMC265183

[10] Martin-Latil S., Gnadig N.F., Mallet A., Desdouits M., Guivel-Benhassine F., Jeannin P. et al. (2012) Transcytosis of HTLV-1 across a tight human epithelial barrier and infection of subepithelial dendritic cells. Blood 120, 572–580 10.1182/blood-2011-08-374637 22589473

[11] Jones K.S., Petrow-Sadowski C., Huang Y.K., Bertolette D.C. and Ruscetti F.W. (2008) Cell-free HTLV-1 infects dendritic cells leading to transmission and transformation of CD4(+) T cells. Nat. Med. 14, 429–436 10.1038/nm1745 18376405

[12] Richardson J.H., Edwards A.J., Cruickshank J.K., Rudge P. and Dalgleish A.G. (1990) In vivo cellular tropism of human T-cell leukemia virus type 1. J. Virol. 64, 5682–5687 10.1128/jvi.64.11.5682-5687.1990 1976827 PMC248630

[13] Melamed A., Laydon D.J., Al Khatib H., Rowan A.G., Taylor G.P. and Bangham C.R. (2015) HTLV-1 drives vigorous clonal expansion of infected CD8(+) T cells in natural infection. Retrovirology 12, 91 10.1186/s12977-015-0221-1 26552867 PMC4640420

[14] Eusebio-Ponce E., Anguita E., Paulino-Ramirez R. and Candel F.J. (2019) HTLV-1 infection: an emerging risk. Pathogenesis, epidemiology, diagnosis and associated diseases. Rev. Esp. Quimioter. 32, 485–496 31648512 PMC6913074

[15] Sodroski J.G., Rosen C.A. and Haseltine W.A. (1984) Trans-acting transcriptional activation of the long terminal repeat of human T lymphotropic viruses in infected cells. Science 225, 381–385 10.1126/science.6330891 6330891

[16] Chen I.S., Slamon D.J., Rosenblatt J.D., Shah N.P., Quan S.G. and Wachsman W. (1985) The x gene is essential for HTLV replication. Science 229, 54–58 10.1126/science.2990037 2990037

[17] Felber B.K., Paskalis H., Kleinman-Ewing C., Wong-Staal F. and Pavlakis G.N. (1985) The pX protein of HTLV-I is a transcriptional activator of its long terminal repeats. Science 229, 675–679 10.1126/science.2992082 2992082

[18] Fujisawa J., Seiki M., Kiyokawa T. and Yoshida M. (1985) Functional activation of the long terminal repeat of human T-cell leukemia virus type I by a trans-acting factor. Proc. Natl. Acad. Sci. U.S.A. 82, 2277–2281 10.1073/pnas.82.8.2277 2986109 PMC397540

[19] Lemasson I., Lewis M.R., Polakowski N., Hivin P., Cavanagh M.H., Thebault S. et al. (2007) Human T-cell leukemia virus type 1 (HTLV-1) bZIP protein interacts with the cellular transcription factor CREB to inhibit HTLV-1 transcription. J. Virol. 81, 1543–1553 10.1128/JVI.00480-06 17151132 PMC1797566

[20] Gaudray G., Gachon F., Basbous J., Biard-Piechaczyk M., Devaux C. and Mesnard J.M. (2002) The complementary strand of the human T-cell leukemia virus type 1 RNA genome encodes a bZIP transcription factor that down-regulates viral transcription. J. Virol. 76, 12813–12822 10.1128/JVI.76.24.12813-12822.2002 12438606 PMC136662

[21] Clerc I., Polakowski N., Andre-Arpin C., Cook P., Barbeau B., Mesnard J.M. et al. (2008) An interaction between the human T cell leukemia virus type 1 basic leucine zipper factor (HBZ) and the KIX domain of p300/CBP contributes to the down-regulation of tax-dependent viral transcription by HBZ. J. Biol. Chem. 283, 23903–23913 10.1074/jbc.M803116200 18599479 PMC3259792

[22] Grassmann R., Berchtold S., Radant I., Alt M., Fleckenstein B., Sodroski J.G. et al. (1992) Role of human T-cell leukemia virus type 1 X region proteins in immortalization of primary human lymphocytes in culture. J. Virol. 66, 4570–4575 10.1128/jvi.66.7.4570-4575.1992 1351105 PMC241270

[23] Matsumoto K., Shibata H., Fujisawa J.I., Inoue H., Hakura A., Tsukahara T. et al. (1997) Human T-cell leukemia virus type 1 Tax protein transforms rat fibroblasts via two distinct pathways. J. Virol. 71, 4445–4451 10.1128/jvi.71.6.4445-4451.1997 9151835 PMC191663

[24] Grossman W.J., Kimata J.T., Wong F.H., Zutter M., Ley T.J. and Ratner L. (1995) Development of leukemia in mice transgenic for the tax gene of human T-cell leukemia virus type I. Proc. Natl. Acad. Sci. U.S.A. 92, 1057–1061 10.1073/pnas.92.4.1057 7862633 PMC42636

[25] Hall A.P., Irvine J., Blyth K., Cameron E.R., Onions D.E. and Campbell M.E. (1998) Tumours derived from HTLV-I tax transgenic mice are characterized by enhanced levels of apoptosis and oncogene expression. J. Pathol. 186, 209–214 10.1002/(SICI)1096-9896(1998100)186:2<209::AID-PATH162>3.0.CO;2-I 9924438

[26] Fujisawa J., Seiki M., Sato M. and Yoshida M. (1986) A transcriptional enhancer sequence of HTLV-I is responsible for trans-activation mediated by p40 chi HTLV-I. EMBO J. 5, 713–718 10.1002/j.1460-2075.1986.tb04272.x 3011423 PMC1166849

[27] Shimotohno K., Takano M., Teruuchi T. and Miwa M. (1986) Requirement of multiple copies of a 21-nucleotide sequence in the U3 regions of human T-cell leukemia virus type I and type II long terminal repeats for trans-acting activation of transcription. Proc. Natl. Acad. Sci. U.S.A. 83, 8112–8116 10.1073/pnas.83.21.8112 3022280 PMC386877

[28] Jeang K.T., Boros I., Brady J., Radonovich M. and Khoury G. (1988) Characterization of cellular factors that interact with the human T-cell leukemia virus type I p40x-responsive 21-base-pair sequence. J. Virol. 62, 4499–4509 10.1128/jvi.62.12.4499-4509.1988 3263510 PMC253560

[29] Baranger A.M., Palmer C.R., Hamm M.K., Giebler H.A., Brauweiler A., Nyborg J.K. et al. (1995) Mechanism of DNA-binding enhancement by the human T-cell leukaemia virus transactivator Tax. Nature 376, 606–608 10.1038/376606a0 7637812

[30] Adya N. and Giam C.Z. (1995) Distinct regions in human T-cell lymphotropic virus type I tax mediate interactions with activator protein CREB and basal transcription factors. J. Virol. 69, 1834–1841 10.1128/jvi.69.3.1834-1841.1995 7853524 PMC188794

[31] Nyborg J.K., Egan D. and Sharma N. (2010) The HTLV-1 Tax protein: revealing mechanisms of transcriptional activation through histone acetylation and nucleosome disassembly. Biochim. Biophys. Acta 1799, 266–274 10.1016/j.bbagrm.2009.09.002 19782779

[32] Lu H., Pise-Masison C.A., Linton R., Park H.U., Schiltz R.L., Sartorelli V. et al. (2004) Tax relieves transcriptional repression by promoting histone deacetylase 1 release from the human T-cell leukemia virus type 1 long terminal repeat. J. Virol. 78, 6735–6743 10.1128/JVI.78.13.6735-6743.2004 15194748 PMC421680

[33] Kamoi K., Yamamoto K., Misawa A., Miyake A., Ishida T., Tanaka Y. et al. (2006) SUV39H1 interacts with HTLV-1 Tax and abrogates Tax transactivation of HTLV-1 LTR. Retrovirology 3, 5 10.1186/1742-4690-3-5 16409643 PMC1363732

[34] Pichler K., Schneider G. and Grassmann R. (2008) MicroRNA miR-146a and further oncogenesis-related cellular microRNAs are dysregulated in HTLV-1-transformed T lymphocytes. Retrovirology 5, 100 10.1186/1742-4690-5-100 19014482 PMC2628945

[35] Fochi S., Ciminale V., Trabetti E., Bertazzoni U., D’Agostino D.M., Zipeto D. et al. (2019) NF-kappaB and microRNA deregulation mediated by HTLV-1 tax and HBZ. Pathogens 8, 4 10.3390/pathogens8040290 31835460 PMC6963194

[36] Van Duyne R., Guendel I., Klase Z., Narayanan A., Coley W., Jaworski E. et al. (2012) Localization and sub-cellular shuttling of HTLV-1 tax with the miRNA machinery. PLoS ONE 7, e40662 10.1371/journal.pone.0040662 22808228 PMC3393700

[37] Henkel T., Zabel U., van Zee K., Muller J.M., Fanning E. and Baeuerle P.A. (1992) Intramolecular masking of the nuclear location signal and dimerization domain in the precursor for the p50 NF-kappa B subunit. Cell 68, 1121–1133 10.1016/0092-8674(92)90083-O 1547506

[38] Chu Z.L., Shin Y.A., Yang J.M., DiDonato J.A. and Ballard D.W. (1999) IKKgamma mediates the interaction of cellular IkappaB kinases with the tax transforming protein of human T cell leukemia virus type 1. J. Biol. Chem. 274, 15297–15300 10.1074/jbc.274.22.15297 10336413

[39] Geleziunas R., Ferrell S., Lin X., Mu Y., Cunningham E.T. Jr, Grant M. et al. (1998) Human T-cell leukemia virus type 1 Tax induction of NF-kappaB involves activation of the IkappaB kinase alpha (IKKalpha) and IKKbeta cellular kinases. Mol. Cell. Biol. 18, 5157–5165 10.1128/MCB.18.9.5157 9710600 PMC109101

[40] Suzuki T., Hirai H. and Yoshida M. (1994) Tax protein of HTLV-1 interacts with the Rel homology domain of NF-kappa B p65 and c-Rel proteins bound to the NF-kappa B binding site and activates transcription. Oncogene 9, 3099–3105 7936632

[41] Wu X. and Sun S.C. (2007) Retroviral oncoprotein Tax deregulates NF-kappaB by activating Tak1 and mediating the physical association of Tak1-IKK. EMBO Rep. 8, 510–515 10.1038/sj.embor.7400931 17363973 PMC1866198

[42] Ho Y.K., Zhi H., Bowlin T., Dorjbal B., Philip S., Zahoor M.A. et al. (2015) HTLV-1 tax stimulates ubiquitin E3 ligase, ring finger protein 8, to assemble lysine 63-linked polyubiquitin chains for TAK1 and IKK activation. PLoS Pathog. 11, e1005102 10.1371/journal.ppat.1005102 26285145 PMC4540474

[43] Fu D.X., Kuo Y.L., Liu B.Y., Jeang K.T. and Giam C.Z. (2003) Human T-lymphotropic virus type I tax activates I-kappa B kinase by inhibiting I-kappa B kinase-associated serine/threonine protein phosphatase 2A. J. Biol. Chem. 278, 1487–1493 10.1074/jbc.M210631200 12419799

[44] Shembade N., Harhaj N.S., Parvatiyar K., Copeland N.G., Jenkins N.A., Matesic L.E. et al. (2008) The E3 ligase Itch negatively regulates inflammatory signaling pathways by controlling the function of the ubiquitin-editing enzyme A20. Nat. Immunol. 9, 254–262 10.1038/ni1563 18246070

[45] Harhaj E.W. and Giam C.Z. (2018) NF-kappaB signaling mechanisms in HTLV-1-induced adult T-cell leukemia/lymphoma. FEBS J. 285, 3324–3336 10.1111/febs.14492 29722927 PMC6750271

[46] Mori N., Fujii M., Ikeda S., Yamada Y., Tomonaga M., Ballard D.W. et al. (1999) Constitutive activation of NF-kappaB in primary adult T-cell leukemia cells. Blood 93, 2360–2368 10090947

[47] Kawakami A., Nakashima T., Sakai H., Urayama S., Yamasaki S., Hida A. et al. (1999) Inhibition of caspase cascade by HTLV-I tax through induction of NF-kappaB nuclear translocation. Blood 94, 3847–3854 10.1182/blood.V94.11.3847 10572100

[48] Kawakami H., Tomita M., Matsuda T., Ohta T., Tanaka Y., Fujii M. et al. (2005) Transcriptional activation of survivin through the NF-kappaB pathway by human T-cell leukemia virus type I tax. Int. J. Cancer 115, 967–974 10.1002/ijc.20954 15729715

[49] Zhang M., Mathews Griner L.A., Ju W., Duveau D.Y., Guha R., Petrus M.N. et al. (2015) Selective targeting of JAK/STAT signaling is potentiated by Bcl-xL blockade in IL-2-dependent adult T-cell leukemia. Proc. Natl. Acad. Sci. U.S.A. 112, 12480–12485 10.1073/pnas.1516208112 26396258 PMC4603455

[50] Choi Y.B. and Harhaj E.W. (2014) HTLV-1 tax stabilizes MCL-1 via TRAF6-dependent K63-linked polyubiquitination to promote cell survival and transformation. PLoS Pathog. 10, e1004458 10.1371/journal.ppat.1004458 25340740 PMC4207805

[51] Chlichlia K. and Khazaie K. (2010) HTLV-1 Tax: linking transformation, DNA damage and apoptotic T-cell death. Chem. Biol. Interact. 188, 359–365 10.1016/j.cbi.2010.06.005 20558150

[52] Rivera-Walsh I., Waterfield M., Xiao G., Fong A. and Sun S.C. (2001) NF-kappaB signaling pathway governs TRAIL gene expression and human T-cell leukemia virus-I Tax-induced T-cell death. J. Biol. Chem. 276, 40385–40388 10.1074/jbc.C100501200 11553609

[53] Chen X., Zachar V., Zdravkovic M., Guo M., Ebbesen P. and Liu X. (1997) Role of the Fas/Fas ligand pathway in apoptotic cell death induced by the human T cell lymphotropic virus type I Tax transactivator. J. Gen. Virol. 78, 3277–3285 10.1099/0022-1317-78-12-3277 9400978

[54] Chlichlia K., Busslinger M., Peter M.E., Walczak H., Krammer P.H., Schirrmacher V. et al. (1997) ICE-proteases mediate HTLV-I Tax-induced apoptotic T-cell death. Oncogene 14, 2265–2272 10.1038/sj.onc.1201070 9178902

[55] Jeong S.J., Radonovich M., Brady J.N. and Pise-Masison C.A. (2004) HTLV-I Tax induces a novel interaction between p65/RelA and p53 that results in inhibition of p53 transcriptional activity. Blood 104, 1490–1497 10.1182/blood-2003-12-4174 15155458

[56] Vajente N., Trevisan R. and Saggioro D. (2009) HTLV-1 Tax protein cooperates with Ras in protecting cells from apoptosis. Apoptosis 14, 153–163 10.1007/s10495-008-0289-3 19089619

[57] Yoshita M., Higuchi M., Takahashi M., Oie M., Tanaka Y. and Fujii M. (2012) Activation of mTOR by human T-cell leukemia virus type 1 Tax is important for the transformation of mouse T cells to interleukin-2-independent growth. Cancer Sci. 103, 369–374 10.1111/j.1349-7006.2011.02123.x 22010857

[58] Tomita M. (2012) Important roles of cellular microRNA miR-155 in leukemogenesis by human T-cell leukemia virus type 1 infection. ISRN Microbiol. 2012, 978607 10.5402/2012/978607 23762762 PMC3671690

[59] Tomita M., Tanaka Y. and Mori N. (2012) MicroRNA miR-146a is induced by HTLV-1 tax and increases the growth of HTLV-1-infected T-cells. Int. J. Cancer 130, 2300–2309 10.1002/ijc.25115 20017139

[60] Blackford A.N. and Jackson S.P. (2017) ATM, ATR, and DNA-PK: the trinity at the heart of the DNA damage response. Mol. Cell 66, 801–817 10.1016/j.molcel.2017.05.015 28622525

[61] Belgnaoui S.M., Fryrear K.A., Nyalwidhe J.O., Guo X. and Semmes O.J. (2010) The viral oncoprotein tax sequesters DNA damage response factors by tethering MDC1 to chromatin. J. Biol. Chem. 285, 32897–32905 10.1074/jbc.M110.146373 20729195 PMC2963403

[62] Chandhasin C., Ducu R.I., Berkovich E., Kastan M.B. and Marriott S.J. (2008) Human T-cell leukemia virus type 1 tax attenuates the ATM-mediated cellular DNA damage response. J. Virol. 82, 6952–6961 10.1128/JVI.02331-07 18434398 PMC2446947

[63] Kibler K.V. and Jeang K.T. (2001) CREB/ATF-dependent repression of cyclin a by human T-cell leukemia virus type 1 Tax protein. J. Virol. 75, 2161–2173 10.1128/JVI.75.5.2161-2173.2001 11160720 PMC114800

[64] Yam C.H., Fung T.K. and Poon R.Y. (2002) Cyclin A in cell cycle control and cancer. Cell. Mol. Life Sci. 59, 1317–1326 10.1007/s00018-002-8510-y 12363035 PMC11337442

[65] Kinjo T., Ham-Terhune J., Peloponese J.M. Jr and Jeang K.T. (2010) Induction of reactive oxygen species by human T-cell leukemia virus type 1 tax correlates with DNA damage and expression of cellular senescence marker. J. Virol. 84, 5431–5437 10.1128/JVI.02460-09 20219913 PMC2863840

[66] Takahashi M., Higuchi M., Makokha G.N., Matsuki H., Yoshita M., Tanaka Y. et al. (2013) HTLV-1 Tax oncoprotein stimulates ROS production and apoptosis in T cells by interacting with USP10. Blood 122, 715–725 10.1182/blood-2013-03-493718 23775713

[67] Ohsugi T., Kumasaka T., Okada S. and Urano T. (2007) The Tax protein of HTLV-1 promotes oncogenesis in not only immature T cells but also mature T cells. Nat. Med. 13, 527–528 10.1038/nm0507-527 17479090

[68] Hasegawa H., Sawa H., Lewis M.J., Orba Y., Sheehy N., Yamamoto Y. et al. (2006) Thymus-derived leukemia-lymphoma in mice transgenic for the Tax gene of human T-lymphotropic virus type I. Nat. Med. 12, 466–472 10.1038/nm1389 16550188

[69] Forlani G., Shallak M., Tedeschi A., Cavallari I., Marçais A., Hermine O. et al. (2021) Dual cytoplasmic and nuclear localization of HTLV-1-encoded HBZ protein is a unique feature of adult T-cell leukemia. Haematologica 106, 2076–2085 10.3324/haematol.2020.272468 33626865 PMC8327710

[70] Mesnard J.M., Barbeau B., Cesaire R. and Peloponese J.M. (2015) Roles of HTLV-1 basic zip factor (HBZ) in viral chronicity and leukemic transformation. Potential new therapeutic approaches to prevent and treat HTLV-1-related diseases. Viruses 7, 6490–6505 10.3390/v7122952 26690203 PMC4690875

[71] Zhao T., Yasunaga J., Satou Y., Nakao M., Takahashi M., Fujii M. et al. (2009) Human T-cell leukemia virus type 1 bZIP factor selectively suppresses the classical pathway of NF-kappaB. Blood 113, 2755–2764 10.1182/blood-2008-06-161729 19064727

[72] Arnold J., Yamamoto B., Li M., Phipps A.J., Younis I., Lairmore M.D. et al. (2006) Enhancement of infectivity and persistence in vivo by HBZ, a natural antisense coded protein of HTLV-1. Blood 107, 3976–3982 10.1182/blood-2005-11-4551 16424388 PMC1895283

[73] Thebault S., Basbous J., Hivin P., Devaux C. and Mesnard J.M. (2004) HBZ interacts with JunD and stimulates its transcriptional activity. FEBS Lett. 562, 165–170 10.1016/S0014-5793(04)00225-X 15044019

[74] Hagiya K., Yasunaga J., Satou Y., Ohshima K. and Matsuoka M. (2011) ATF3, an HTLV-1 bZip factor binding protein, promotes proliferation of adult T-cell leukemia cells. Retrovirology 8, 19 10.1186/1742-4690-8-19 21414204 PMC3068935

[75] Gazon H., Barbeau B., Mesnard J.M. and Peloponese J.M. Jr (2017) Hijacking of the AP-1 signaling pathway during development of ATL. Front. Microbiol. 8, 2686 10.3389/fmicb.2017.02686 29379481 PMC5775265

[76] Gazon H., Lemasson I., Polakowski N., Cesaire R., Matsuoka M., Barbeau B. et al. (2012) Human T-cell leukemia virus type 1 (HTLV-1) bZIP factor requires cellular transcription factor JunD to upregulate HTLV-1 antisense transcription from the 3′ long terminal repeat. J. Virol. 86, 9070–9078 10.1128/JVI.00661-12 22696638 PMC3416116

[77] Nakayama T., Higuchi T., Oiso N., Kawada A. and Yoshie O. (2012) Expression and function of FRA2/JUND in cutaneous T-cell lymphomas. Anticancer Res. 32, 1367–1373 22493372

[78] Satou Y., Yasunaga J., Yoshida M. and Matsuoka M. (2006) HTLV-I basic leucine zipper factor gene mRNA supports proliferation of adult T cell leukemia cells. Proc. Natl. Acad. Sci. U.S.A. 103, 720–725 10.1073/pnas.0507631103 16407133 PMC1334651

[79] Vernin C., Thenoz M., Pinatel C., Gessain A., Gout O., Delfau-Larue M.H. et al. (2014) HTLV-1 bZIP factor HBZ promotes cell proliferation and genetic instability by activating OncomiRs. Cancer Res. 74, 6082–6093 10.1158/0008-5472.CAN-13-3564 25205102

[80] Tanaka-Nakanishi A., Yasunaga J., Takai K. and Matsuoka M. (2014) HTLV-1 bZIP factor suppresses apoptosis by attenuating the function of FoxO3a and altering its localization. Cancer Res. 74, 188–200 10.1158/0008-5472.CAN-13-0436 24177179

[81] Nicot C., Harrod R.L., Ciminale V. and Franchini G. (2005) Human T-cell leukemia/lymphoma virus type 1 nonstructural genes and their functions. Oncogene 24, 6026–6034 10.1038/sj.onc.1208977 16155609

[82] Nicot C., Dundr M., Johnson J.M., Fullen J.R., Alonzo N., Fukumoto R. et al. (2004) HTLV-1-encoded p30II is a post-transcriptional negative regulator of viral replication. Nat. Med. 10, 197–201 10.1038/nm984 14730358

[83] Zhang W., Nisbet J.W., Albrecht B., Ding W., Kashanchi F., Bartoe J.T. et al. (2001) Human T-lymphotropic virus type 1 p30(II) regulates gene transcription by binding CREB binding protein/p300. J. Virol. 75, 9885–9895 10.1128/JVI.75.20.9885-9895.2001 11559821 PMC114560

[84] Malu A., Hutchison T., Yapindi L., Smith K., Nelson K., Bergeson R. et al. (2019) The human T-cell leukemia virus type-1 tax oncoprotein dissociates NF-κB p65(RelA)-Stathmin complexes and causes catastrophic mitotic spindle damage and genomic instability. Virology 535, 83–101 10.1016/j.virol.2019.07.003 31299491 PMC6940561

[85] Awasthi S., Sharma A., Wong K., Zhang J., Matlock E.F., Rogers L. et al. (2005) A human T-cell lymphotropic virus type 1 enhancer of Myc transforming potential stabilizes Myc-TIP60 transcriptional interactions. Mol. Cell. Biol. 25, 6178–6198 10.1128/MCB.25.14.6178-6198.2005 15988028 PMC1168837

[86] Andresen V., Pise-Masison C.A., Sinha-Datta U., Bellon M., Valeri V., Washington Parks R. et al. (2011) Suppression of HTLV-1 replication by Tax-mediated rerouting of the p13 viral protein to nuclear speckles. Blood 118, 1549–1559 10.1182/blood-2010-06-293340 21677314 PMC3156045

[87] Silic-Benussi M., Cavallari I., Vajente N., Vidali S., Chieco-Bianchi L., Di Lisa F. et al. (2010) Redox regulation of T-cell turnover by the p13 protein of human T-cell leukemia virus type 1: distinct effects in primary versus transformed cells. Blood 116, 54–62 10.1182/blood-2009-07-235861 20395415

[88] Silic-Benussi M., Cannizzaro E., Venerando A., Cavallari I., Petronilli V., La Rocca N. et al. (2009) Modulation of mitochondrial K(+) permeability and reactive oxygen species production by the p13 protein of human T-cell leukemia virus type 1. Biochim. Biophys. Acta 1787, 947–954 10.1016/j.bbabio.2009.02.001 19366603

[89] Nicot C., Mulloy J.C., Ferrari M.G., Johnson J.M., Fu K., Fukumoto R. et al. (2001) HTLV-1 p12(I) protein enhances STAT5 activation and decreases the interleukin-2 requirement for proliferation of primary human peripheral blood mononuclear cells. Blood 98, 823–829 10.1182/blood.V98.3.823 11468184

[90] Ding W., Albrecht B., Luo R., Zhang W., Stanley J.R., Newbound G.C. et al. (2001) Endoplasmic reticulum and cis-Golgi localization of human T-lymphotropic virus type 1 p12(I): association with calreticulin and calnexin. J. Virol. 75, 7672–7682 10.1128/JVI.75.16.7672-7682.2001 11462039 PMC115002

[91] Albrecht B., Collins N.D., Burniston M.T., Nisbet J.W., Ratner L., Green P.L. et al. (2000) Human T-lymphotropic virus type 1 open reading frame I p12(I) is required for efficient viral infectivity in primary lymphocytes. J. Virol. 74, 9828–9835 10.1128/JVI.74.21.9828-9835.2000 11024109 PMC102019

[92] Lairmore M.D., Albrecht B., D'Souza C., Nisbet J.W., Ding W., Bartoe J.T. et al. (2000) In vitro and in vivo functional analysis of human T cell lymphotropic virus type 1 pX open reading frames I and II. AIDS Res. Hum. Retroviruses 16, 1757–1764 10.1089/08892220050193272 11080823

[93] Collins N.D., Newbound G.C., Albrecht B., Beard J.L., Ratner L. and Lairmore M.D. (1998) Selective ablation of human T-cell lymphotropic virus type 1 p12I reduces viral infectivity in vivo. Blood 91, 4701–4707 10.1182/blood.V91.12.4701.412k23_4701_4707 9616168

[94] Takeda S., Maeda M., Morikawa S., Taniguchi Y., Yasunaga J., Nosaka K. et al. (2004) Genetic and epigenetic inactivation of tax gene in adult T-cell leukemia cells. Int. J. Cancer 109, 559–567 10.1002/ijc.20007 14991578

[95] Nosaka K. and Matsuoka M. (2021) Adult T-cell leukemia-lymphoma as a viral disease: subtypes based on viral aspects. Cancer Sci. 112, 1688–1694 10.1111/cas.14869 33630351 PMC8088923

[96] Kataoka K., Nagata Y., Kitanaka A., Shiraishi Y., Shimamura T., Yasunaga J. et al. (2015) Integrated molecular analysis of adult T cell leukemia/lymphoma. Nat. Genet. 47, 1304–1315 10.1038/ng.3415 26437031

[97] Shigemura T., Shiohara M., Kato M., Furuta S., Kaneda K., Morishita K. et al. (2015) Superoxide-generating Nox5alpha is functionally required for the human T-cell leukemia virus type 1-induced cell transformation phenotype. J. Virol. 89, 9080–9089 10.1128/JVI.00983-15 26109726 PMC4524067

[98] Pan F., Ye Z., Cheng L. and Liu J.O. (2004) Myocyte enhancer factor 2 mediates calcium-dependent transcription of the interleukin-2 gene in T lymphocytes: a calcium signaling module that is distinct from but collaborates with the nuclear factor of activated T cells (NFAT). J. Biol. Chem. 279, 14477–14480 10.1074/jbc.C300487200 14722108

[99] Jain P., Lavorgna A., Sehgal M., Gao L., Ginwala R., Sagar D. et al. (2015) Myocyte enhancer factor (MEF)-2 plays essential roles in T-cell transformation associated with HTLV-1 infection by stabilizing complex between Tax and CREB. Retrovirology 12, 23 10.1186/s12977-015-0140-1 25809782 PMC4374383

[100] Yan P., Fu J., Qu Z., Li S., Tanaka T., Grusby M.J. et al. (2009) PDLIM2 suppresses human T-cell leukemia virus type I Tax-mediated tumorigenesis by targeting Tax into the nuclear matrix for proteasomal degradation. Blood 113, 4370–4380 10.1182/blood-2008-10-185660 19131544 PMC2676091

[101] Yan P., Qu Z., Ishikawa C., Mori N. and Xiao G. (2009) Human T-cell leukemia virus type I-mediated repression of PDZ-LIM domain-containing protein 2 involves DNA methylation but independent of the viral oncoprotein tax. Neoplasia 11, 1036–1041 10.1593/neo.09752 19794962 PMC2745669

[102] Nadella M.V., Shu S.T., Dirksen W.P., Thudi N.K., Nadella K.S., Fernandez S.A. et al. (2008) Expression of parathyroid hormone-related protein during immortalization of human peripheral blood mononuclear cells by HTLV-1: implications for transformation. Retrovirology 5, 46 10.1186/1742-4690-5-46 18541021 PMC2435116

[103] Watanabe T., Yamaguchi K., Takatsuki K., Osame M. and Yoshida M. (1990) Constitutive expression of parathyroid hormone-related protein gene in human T cell leukemia virus type 1 (HTLV-1) carriers and adult T cell leukemia patients that can be trans-activated by HTLV-1 tax gene. J. Exp. Med. 172, 759–765 10.1084/jem.172.3.759 2388034 PMC2188541

[104] Panfil A.R., Al-Saleem J., Howard C.M., Mates J.M., Kwiek J.J., Baiocchi R.A. et al. (2015) PRMT5 is upregulated in HTLV-1-mediated T-cell transformation and selective inhibition alters viral gene expression and infected cell survival. Viruses 8, 1 10.3390/v8010007 26729154 PMC4728567

[105] Ruggero K., Corradin A., Zanovello P., Amadori A., Bronte V., Ciminale V. et al. (2010) Role of microRNAs in HTLV-1 infection and transformation. Mol. Aspects Med. 31, 367–382 10.1016/j.mam.2010.05.001 20600265

[106] Petrocca F., Visone R., Onelli M.R., Shah M.H., Nicoloso M.S., de Martino I. et al. (2008) E2F1-regulated microRNAs impair TGFbeta-dependent cell-cycle arrest and apoptosis in gastric cancer. Cancer Cell 13, 272–286 10.1016/j.ccr.2008.02.013 18328430

[107] Stern-Ginossar N., Gur C., Biton M., Horwitz E., Elboim M., Stanietsky N. et al. (2008) Human microRNAs regulate stress-induced immune responses mediated by the receptor NKG2D. Nat. Immunol. 9, 1065–1073 10.1038/ni.1642 18677316

[108] Yeung M.L., Yasunaga J., Bennasser Y., Dusetti N., Harris D., Ahmad N. et al. (2008) Roles for microRNAs, miR-93 and miR-130b, and tumor protein 53-induced nuclear protein 1 tumor suppressor in cell growth dysregulation by human T-cell lymphotrophic virus 1. Cancer Res. 68, 8976–8985 10.1158/0008-5472.CAN-08-0769 18974142 PMC2596768

[109] Chang T.C., Yu D., Lee Y.S., Wentzel E.A., Arking D.E., West K.M. et al. (2008) Widespread microRNA repression by Myc contributes to tumorigenesis. Nat. Genet. 40, 43–50 10.1038/ng.2007.30 18066065 PMC2628762

[110] Anderson M.D., Ye J., Xie L. and Green P.L. (2004) Transformation studies with a human T-cell leukemia virus type 1 molecular clone. J. Virol. Methods 116, 195–202 10.1016/j.jviromet.2003.11.016 14738988

[111] Yin H., Kannian P., Dissinger N., Haines R., Niewiesk S. and Green P.L. (2012) Human T-cell leukemia virus type 2 antisense viral protein 2 is dispensable for in vitro immortalization but functions to repress early virus replication in vivo. J. Virol. 86, 8412–8421 10.1128/JVI.00717-12 22623800 PMC3421770

[112] Xie L., Yamamoto B., Haoudi A., Semmes O.J. and Green P.L. (2006) PDZ binding motif of HTLV-1 Tax promotes virus-mediated T-cell proliferation in vitro and persistence in vivo. Blood 107, 1980–1988 10.1182/blood-2005-03-1333 16263794 PMC1895710

[113] Martinez M.P., Cheng X., Joseph A., Al-Saleem J., Panfil A.R., Palettas M. et al. (2019) HTLV-1 CTCF-binding site is dispensable for in vitro immortalization and persistent infection in vivo. Retrovirology 16, 44 10.1186/s12977-019-0507-9 31864373 PMC6925871

[114] Nerenberg M., Hinrichs S.H., Reynolds R.K., Khoury G. and Jay G. (1987) The tat gene of human T-lymphotropic virus type 1 induces mesenchymal tumors in transgenic mice. Science 237, 1324–1329 10.1126/science.2888190 2888190

[115] Benvenisty N., Ornitz D.M., Bennett G.L., Sahagan B.G., Kuo A., Cardiff R.D. et al. (1992) Brain tumours and lymphomas in transgenic mice that carry HTLV-I LTR/c-myc and Ig/tax genes. Oncogene 7, 2399–2405 1461648

[116] Iwakura Y., Saijo S., Kioka Y., Nakayama-Yamada J., Itagaki K., Tosu M. et al. (1995) Autoimmunity induction by human T cell leukemia virus type 1 in transgenic mice that develop chronic inflammatory arthropathy resembling rheumatoid arthritis in humans. J. Immunol. 155, 1588–1598 7636219

[117] Rauch D., Gross S., Harding J., Bokhari S., Niewiesk S., Lairmore M. et al. (2009) T-cell activation promotes tumorigenesis in inflammation-associated cancer. Retrovirology 6, 116 10.1186/1742-4690-6-116 20017942 PMC2806367

[118] Rauch D., Gross S., Harding J., Niewiesk S., Lairmore M., Piwnica-Worms D. et al. (2009) Imaging spontaneous tumorigenesis: inflammation precedes development of peripheral NK tumors. Blood 113, 1493–1500 10.1182/blood-2008-07-166462 18971418 PMC2644076

[119] Watters K.M., Dean J., Hasegawa H., Sawa H., Hall W. and Sheehy N. (2010) Cytokine and growth factor expression by HTLV-1 Lck-tax transgenic cells in SCID mice. AIDS Res. Hum. Retroviruses 26, 593–603 10.1089/aid.2009.0212 20438380

[120] Satou Y., Yasunaga J., Zhao T., Yoshida M., Miyazato P., Takai K. et al. (2011) HTLV-1 bZIP factor induces T-cell lymphoma and systemic inflammation in vivo. PLoS Pathog. 7, e1001274 10.1371/journal.ppat.1001274 21347344 PMC3037353

[121] Ishihara S., Tachibana N., Okayama A., Murai K., Tsuda K. and Mueller N. (1992) Successful graft of HTLV-I-transformed human T-cells (MT-2) in severe combined immunodeficiency mice treated with anti-asialo GM-1 antibody. Jpn. J. Cancer Res. 83, 320–323 10.1111/j.1349-7006.1992.tb00108.x 1506264 PMC5918837

[122] Feuer G., Zack J.A., Harrington W.J. Jr, Valderama R., Rosenblatt J.D., Wachsman W. et al. (1993) Establishment of human T-cell leukemia virus type I T-cell lymphomas in severe combined immunodeficient mice. Blood 82, 722–731 10.1182/blood.V82.3.722.722 8338942

[123] Dewan M.Z., Terashima K., Taruishi M., Hasegawa H., Ito M., Tanaka Y. et al. (2003) Rapid tumor formation of human T-cell leukemia virus type 1-infected cell lines in novel NOD-SCID/gammac(null) mice: suppression by an inhibitor against NF-kappaB. J. Virol. 77, 5286–5294 10.1128/JVI.77.9.5286-5294.2003 12692230 PMC153944

[124] Arnold J., Zimmerman B., Li M., Lairmore M.D. and Green P.L. (2008) Human T-cell leukemia virus type-1 antisense-encoded gene, Hbz, promotes T-lymphocyte proliferation. Blood 112, 3788–3797 10.1182/blood-2008-04-154286 18689544 PMC2572803

[125] Huey D.D., Bolon B., La Perle K.M.D., Kannian P., Jacobson S., Ratner L. et al. (2018) Role of wild-type and recombinant human T-cell leukemia viruses in lymphoproliferative disease in humanized NSG mice. Comp. Med. 68, 4–14 29460716 PMC5824134

[126] Huey D.D. and Niewiesk S. (2018) Production of humanized mice through stem cell transfer. Curr. Protoc. Mouse Biol. 8, 17–27 10.1002/cpmo.38 29988984 PMC6034996

[127] Tezuka K., Xun R., Tei M., Ueno T., Tanaka M., Takenouchi N. et al. (2014) An animal model of adult T-cell leukemia: humanized mice with HTLV-1-specific immunity. Blood 123, 346–355 10.1182/blood-2013-06-508861 24196073

[128] Shirinian M., Kambris Z., Hamadeh L., Grabbe C., Journo C., Mahieux R. et al. (2015) A transgenic Drosophila melanogaster model to study human T-lymphotropic virus oncoprotein Tax-1-driven transformation in vivo. J. Virol. 89, 8092–8095 10.1128/JVI.00918-15 25995252 PMC4505646

[129] Akkouche A., Moodad S., Hleihel R., Skayneh H., Chambeyron S., El Hajj H. et al. (2021) In vivo antagonistic role of the human T-cell leukemia virus type 1 regulatory proteins Tax and HBZ. PLoS Pathog. 17, e1009219 10.1371/journal.ppat.1009219 33471856 PMC7817025

[130] Yin H., Kannian P., Dissinger N., Haines R., Niewiesk S. and Green P.L. (2012) HTLV-2 APH-2 is dispensable for in vitro immortalization, but functions to repress early viral replication in vivo. J. Virol. 86, 8412–8421 10.186128/JVI.00717-12 22623800 PMC3421770

